# Melatonin mediates monochromatic green light-induced satellite cell proliferation and muscle growth in chick embryo

**DOI:** 10.1371/journal.pone.0216392

**Published:** 2019-05-06

**Authors:** Xinjie Bai, Jing Cao, Yulan Dong, Zixu Wang, Yaoxing Chen

**Affiliations:** 1 Laboratory of Anatomy of Domestic Animals, College of Veterinary Medicine, China Agricultural University, Haidian, Beijing, China; 2 Department of Animal Husbandry and Veterinary, Beijing Vocational College of Agriculture, Beijing, China; University of Minnesota Medical Center, UNITED STATES

## Abstract

**Background:**

Green light penetrates the skull and has directly affected on the secretion of melatonin in plasma, which regulates the endocrine activities to influence the muscle growth, satellite cell mitotic activity and quality properties of meat from the embryonic period to posthatch in chick. Pituitary adenylate cyclase-activating polypeptide 6–38 (PACAP6-38) could inhibit the synthesis and secretion of pineal melatonin. Finding a new way for exploring the mechanism of light-regulated muscle growth in ovo is essential for promoting the productive performance in poultry.

**Methods:**

Chick embryos were exposed to darkness (D-group) and green light (G-group) throughout the embryonic period, and injected with PACAP6-38 or saline at embryonic day 8. Plasma hormone, skeletal muscle fiber areas, satellite cell proliferation activity, paired domain homeobox transcription factor 7 and myogenic regulatory factors were observed.

**Results:**

By saline treatment, the percentage of proliferating cell nuclear antigen immunoreactive cells and mitotic activity of satellite cells in skeletal muscle were higher in G-group than those of in D-group at post-hatching day 0. With the increase of plasma melatonin, green light promoted the secretion of growth hormone (GH) and insulin like factor 1 (IGF-1) in plasma, the satellite cell proliferation, the size of muscle fiber, as well as the mRNA expressions of *Pax7*, *myogenic regulatory factors* and *IGF-1R*. After PACAP6-38 treatment to inhibit the secretion of melatonin in ovo, aforementioned parameters were remarkably decreased and the difference of these parameters was disappeared between D-group and G-group.

**Conclusion:**

These data indicated that stimulation with monochromatic green light during incubation enhanced the secretion of melatonin and up-regulation of GH-IGF-1 axis to activate the satellite cells proliferation and myofiber formation, involving the expression of *Pax7* and *myogenic regulatory factors*.

## Introduction

Birds are very sensitive to detecting a broader spectrum of colors, which is one of the most important environmental factors [1]. Light penetrate the skull and have direct effects on avian pineal gland or hypothalamus [2, 3], which regulate the endocrine activities to affect the physiological functions, including muscle growth [4], morphogenesis of interlimb stepping [5], satellite cell mitotic activity [6] and quality properties of meat [7]. Meanwhile, monochromatic green light influences the satellite cell proliferation activity, number, myofiber areas and muscle mass from the embryonic period in chick [8–10]. However, how monochromatic light affect the satellite cell proliferation is unclear.

Satellite cells are the primary contributors to muscle growth and regeneration [11–13]. The proliferation of satellite cells is a complicated dynamic process which is affected by growth hormone (GH) [14, 15]. Accumulated evidences have shown that genetic networks are involved in myogenesis [16–18]. Satellite cells express the paired domain homeobox transcription factor 7 (Pax7), which is essential for maintenance of adult skeletal muscle satellite cells and skeletal muscle regeneration [19]. Transcription factor myogenic factor 5 (MYF5) and myoblast determination protein (MYOD) are up-regulated in the activation of satellite cells. And the up-regulation of muscle-specific regulatory factor 4 (MRF4) and myogenin are involved in myoblast differentiation. These myogenic regulatory factors (MRFs) determine the myogenic capacity of muscle progenitors in transcription and epigenetics [16]. Our preliminary research have shown that in ovo exposure to monochromatic lights affected late-embryonic [10] and posthatch [8] muscle growth and satellite cell proliferation by insulin like factor 1 (IGF-1) signaling. However, how light information affects the satellite cell proliferation during the chick embryo development is not fully understood.

Miyata et al. have isolated pituitary adenylate cyclase-activating polypeptide (PACAP) from the ovine hypothalamus [20]. PACAP is a 27- or 38- amino acid neuropeptide, which belongs to the vasoactive intestinal polypeptide (VIP)—glucagon—growth hormone releasing hormone (GHRH)—secretin super family [21, 22]. PACAP stimulates cAMP formation and regulates the synthesis and secretion of pineal melatonin in mice [23], rat [24, 25] and chick [26–28]. In bird, PACAP is mainly distributed in diencephalon, brain stem, telencephalon, tectum and cerebellum [29]. Chick embryo has already expressed PACAP mRNA at embryonic days 3.5 (E3.5) [30]. PACAP content in the suprachiasmatic nucleus may be changed by lighting conditions in rat [31]. Moreover, PACAP6-38 (a PACAP antagonist) has the ability to inhibit the functions of PACAP [22, 27]. In addition, treatment with PACAP6-38 in ovo at E8 has an effect on motor activity and social behavior in chicken [32, 33]. However, few studies have reported the effect of PACAP on avian muscle growth, satellite cell proliferation and secretion of IGF-1. Wang et al. have considered that monochromatic light affects the secretion of IGF-1 via the anti-oxidation pathway and JAK2/STAT3 signaling in chick embryo liver, which involve melatonin and melatonin receptor Mel1c [34, 35]. Therefore, exploring the effect of PACAP6-38 on muscle growth of broilers provides a new way for further clarifying the mechanism of light-regulated growth in bird.

## Materials and methods

### Animal treatment

Fertilized eggs of Arbor Acre fertile broiler chicken obtained from a local hatchery (Beijing Huadu Breeding Co, Beijing, China) were weighed and selected with an average of 65 g (range from 63 to 67g). The eggs were randomly divided into two experimental groups: darkness (D-group) and green light (560 nm, G-group, light intensity was 15 lx). Continuous green light was measured with a digital luxmeter (Mastech MS6610, Precision Mastech Enterprises, Hong Kong, China) and was applied throughout the whole incubation period. Incubation conditions were set at a temperature of 37.5 ± 0.1°C and a relative humidity of 60%. The egg turning frequency was 12 times a day, every 2 hours turning once and every time lasting for 3 min.

**Fig 1:**
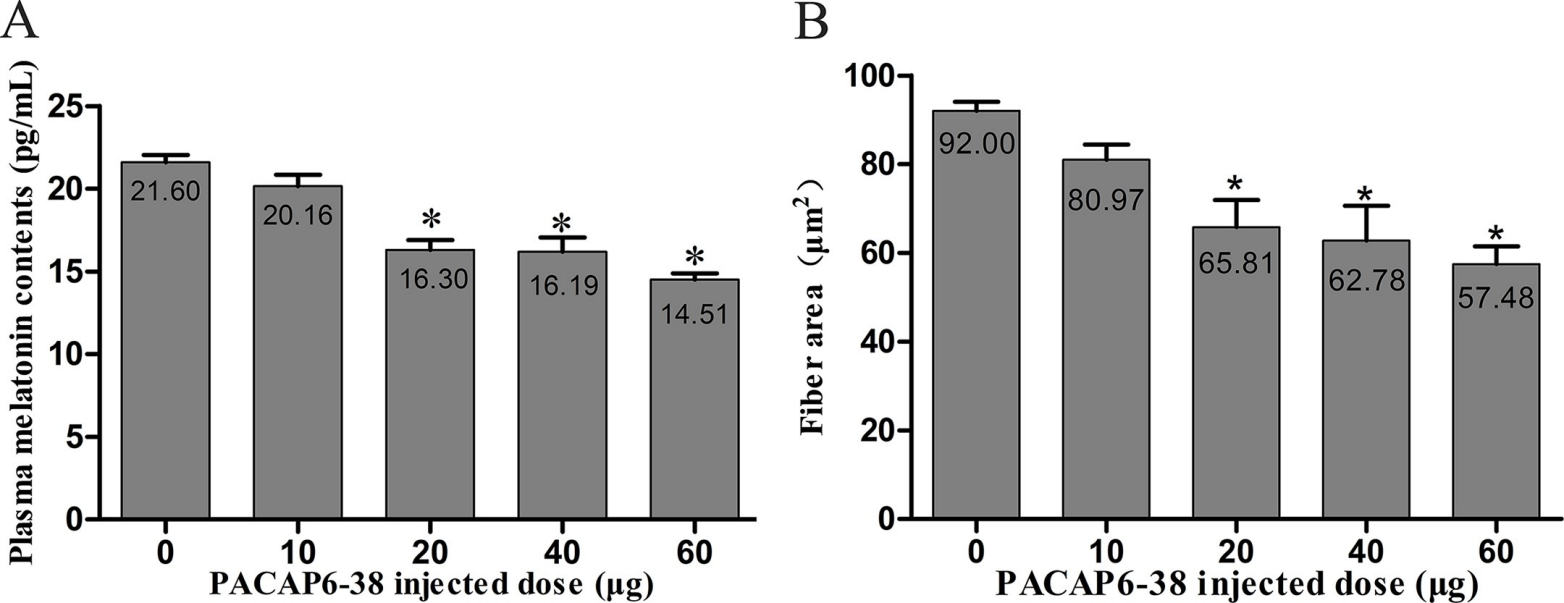
Effect of various doses of PACAP6-38 on plasma melatonin contents and myofiber area. **(A)** A dose-dependent decreased (0, 10, 20, 40 and 60 ng) in plasma melatonin contents with PACAP6-38 treatment. **(B)** A dose-dependent decreased (0, 10, 20, 40 and 60 ng) in the fiber area with PACAP6-38 treatment. Data are mean ± SEM. * p < 0.05 Vs. 0 μg PACAP6-38 injected dose.

The process and the dose of PACAP 6–38 injection were reference to the earlier descriptions [32, 36]. At the end of E8, following candling to locate the position of the air cell, the eggs were disinfected with 75% ethanol and a small window was made on the eggshell with a sterile needle. Meanwhile, the concentrations of PACAP6-38 (0 μg, 10 μg, 20 μg, 40 μg and 60 μg per egg; n = 6, a total of 30) were explored to eliminate the differences between the individuals at E8 in D-group, showed in Fig 1. The results showed that 20 μg/egg of PACAP6-38 as the best concentration had significantly inhibited the secretion of melatonin and the growth of pectoral muscle fiber. Furthermore, a total of 48 eggs included two experimental groups of D-group and G-group. Each group was administrated with 20 μg PACAP6-38 dissolved in 25 μL physiological saline (n = 12) and only with 25 μL saline (n = 12) at E8. After injection, the hole was cellotaped with wax. Chicken embryos went on incubating until posthatch. All experimental procedures were approved by the Animal Welfare Committee of Agricultural Research Organization, China Agricultural University.

### Sample collection

Upon hatching, at post-hatching day 0 (P0), each chick from each group was weighed (BW, g). A total of 2 mL of blood was obtained from the hearts, and plasma was collected from the blood samples for ELISA assay.

Birds were anaesthetized with Nembutal (30–40 mg/g). The major pectoral muscle and the gastrocnemius muscle were taken and weighted. Both indexes of pectoral muscle and gastrocnemius muscle were recorded [organ index = muscle mass weight (g)/ BW (g) ×100%]. Two parts of each muscle sample were taken. One part was fixed in 4% paraformaldehyde in 0.1 M phosphate buffer solution (PB, pH 7.4, 4°C) for 48 h, and the other part was stored in -80°C refrigerators. The samplings were analyzed by the methods of haematoxylin eosin staining (HE), immunohistochemical staining and RT-PCR.

### Measurement of myofiber area

At least 25 random fields in five HE-stained sections of each muscle sample were photographed using a ×40 objective on a microscope (BX51, Olympus, Tokyo, Japan), and 100 fibers per field and a total of 15000 fibers (6 chicks) per treatment were analyzed. Myofiber area was then determined using Image-Pro plus 6.0 software (Media Cybernetics, Inc. Rockville, USA).

### Immunohistochemical staining for PCNA

The paraffin sections of muscle sample were immunohistochemically stained for proliferating cell nuclear antigen (PCNA). Sections were incubated overnight at 4°C with the primary antibody (mouse anti-PCNA, Abcam, 1:2000). Then, the sections were rinsed incubated with biotinylated donkey anti-mouse IgG (1:300, CoWin Biotech Co Inc., Beijing, China) for 2 h at 25°C. After being washed, the tissues were incubated with streptavidin-horseradish peroxidase (1:300, Vector Laboratories, Burlingame, CA, USA) for 2 h at 25°C. Immunoreactivity was visualized by 0.05% 3’3-diaminobenzidine tetrahydrochloride (DAB, Sigma) and 0.003% hydrogen peroxide in 0.01 M PBS for 10 min. Nucleus were counterstained with hematoxylin. The positive cells were present yellow-brown staining in the nucleus. The percentage of PCNA-positive nuclei of the total nuclei was counted in 5 random fields of each section under a ×40 objective on a microscope (BX51, Olympus, Tokyo, Japan). A total of 150 fields (6 chicks) per treatment were observed.

### Satellite cell isolation and preparation

Tissues (major pectoral muscle) were taken from 6 chicks in each group. Satellite cells were obtained using a previously described method [6]. The tissues were incubated with collagenase 1 (Sigma, Saint-Quentin Fallavier, France, 1 g/L) for 30 min and then with trypsin (2.5 g/L) for 20 min. Subsequently, satellite cells were collected by density gradient centrifugation at 200 g for 10 min and cultured in Dulbecco’s Modified Eagle’s Medium (DMEM: complete media; Gibco BRL, Grand Island, NY, USA) supplemented with 10% (vol/vol) heat-inactivated fetal calf serum, 100 IU/mL penicillin, 100 mg/mL streptomycin, and 25 mM HEPES buffer (Sigma, USA). Then, the satellite cells were filtered through a mesh (200 μm). The pure satellite cells were obtained from the pectoral muscle using the differential adherent method. Cells viability (> 95%) was estimated by trypan blue exclusion test, and seeded at a density of 1×10^5^ cells/cm^2^ in six-well plates coated with carry sheet glass and 96-well microtitre plates (Costar 3599, Corning Inc., Corning, NY, USA) at 37°C in a 5% CO_2_ incubator. Immunofluorescences for desmin and Pax7 were used to identify the satellite cells, and more than 95% of the cells we obtained were satellite cells.

### Satellite cell proliferation assay

Satellite cell proliferation was measured with the methyl thiazolyl tetrazolium (MTT) assay, a previously described method [6, 8, 37]. Satellite cells were cultured in 6 repetitions into 96-well microtiter plates in an incubator (37°C, 5% CO_2_) for 66 h. Subsequently, 10 μL of 3-(4, 5-dimethylthiazol-2-yl)-2, 5-diphenyl tetrazolium diluted to a concentration of 5 mg/mL was added to each well, and then incubated for 4 h continually. 100 μL of 10% SDS was added in a 0.04 M HCl solution to lyse the cells and solubilize the MTT crystals. After 30 min, the OD value (570 nm) of each sample was detected using an automated microplate reader (Bio-Rad Inc., St Louis, MO, USA).

### ELISA assay

The plasma melatonin, GH and IGF-1 were measured using commercial chicken-specific ELISA kits (melatonin: SEA908Ga; GH: SEA044Ga; IGF-1: SEA050Ga; Cloud-Clone Corp., Houston, TX) according to the manufacturer’s protocol.

The kit for melatonin in gallus plasma is a competitive inhibition enzyme immunoassay. The detection range was 12.35–1000 pg/mL, the intra-assay CV was 9.7%, and the inter-assay CV was 11.2%. The kits for GH and IGF-1 in gallus plasma are sandwich enzyme immunoassay. The detection range was 0.156–10 ng/mL and 0.7–25 ng/mL, the intra-assay CV was 6.1% and 7.9%, and the inter-assay CV was 7.5% and 8.5%, respectively.

### RT-PCR

Total RNA extraction was performed by using TRIzol (CW0580, CwBiotech Co., Inc., Beijing, China). The isolated total RNA was resuspended in 20 μL of diethylpyrocarbonate-treated water, and quality-checked by NanoPhotometer UV/Visible spectrophotometer (Pearl 330, Implen, Germany). A reverse transcription (RT) kit (Thermo Fisher Scientific, USA) was used for cDNA synthesis. RT reactions were performed for 1 h at 42°C containing 2 μg of total RNA and oligodT_18_ primers 1 μL, and superscript reverse transcriptase 1 μL in a final volume of 20 μL for standard PCR. PCR amplification with Taq DNA polymerase was performed as follows: 94°C for 30 s, 56°C for 30 s, and 72°C for 30 s. The PCR primers are listed in Table 1. According to standard techniques, the PCR products were analyzed by electrophoresis on a 2% agarose gel in Tris-acetate-EDTA buffer. The data are shown as the integral optical density (IOD) of the bands normalized to the IOD of the corresponding glyceraldehydes-3-phosphate dehydrogenase (GAPDH) by the image analysis software (Gel-pro analyzer 4.5, Media Cybernetics Inc., Bethesda, MD, USA), and the results were obtained from three separate experiments.

**Table 1:** Primer sequences for RT-PCR analysis.

Genes	Primer sequences	Fragment size (bp)	Cycles	GenBank number
*Pax7*	F: GTGATTCAGCAACCGACGAG R: CATGGTGGATGGTGGCAAG	214	30	NM_001303185.1
*MyoD*	F: CTACAGCGGGGAGTCAGATG R: CCCATGCTTTGGGTCATTTGG	148	34	NM_204214.1
*myogenin*	F: GGCTTTGGAGGAGAAGGACT R: CAGAGTGCTGCGTTTCAG	184	34	NM_204184
*Myf5*	F: CAACCCCAACCAGAGACTCC R: TCCCGGCAGGTGATAGTAGT	115	32	NM_001030363.1
*IGF-1R*	F: CTGTGTCCGACAAATGGGGA R: TGACGGTCAGTTTCGGGAAG	169	33	NM_205032.1
*GAPDH*	F: ATCACAGCCACACAGAAGACG R: TGACTTTCCCCACAGCCTTA	124	27	NM_204305

F = forward primer; R = reverse primer.

### Statistical analysis

The data are presented as mean ± SEM. Differences among treatments were examined by one-way and two-way ANOVA followed by Tukey HSD post hoc test with R version 3.5.2 (R Core Team, Vienna, Austria). Using Pearson’s correlation coefficient with GraphPad prim 5, the correlation among the plasma melatonin and myofiber area, melatonin and GH, GH and IGF-1, melatonin and IGF-1, and the absolute and relative numbers of satellite cells were analyzed, respectively. Values of p < 0.05 were considered significant difference.

## Results

### Determination of PACAP6-38 injected dose

Effects of different concentrations of PACAP6-38 on plasma melatonin and myofiber area were shown in Fig 1. A reduction of plasma melatonin concentrations responded to PACAP6-38 in a dose-dependent fashion was observed (F = 23.624, Fig 1B). Compared with control saline group, the administration of exogenous PACAP6-38 decreased the plasma melatonin (6.67% in 10 μg group, p = 0.478; 24.50% in 20 μg group, p < 0.05; 25.049% in 40 μg group, p < 0.05; and 32.84% in 60 μg group, p < 0.05) in a dose-dependent manner. No significant differences were observed among 20 μg, 40 μg and 60 μg group (p > 0.05). A similar change was found that injection of exogenous PACAP6-38 induced a decrease of myofiber area in a dose-dependent fashion (F = 7.746, Fig 1A). Interestingly, the myofiber area was significantly decreased in 20 μg group than that of in control group (28.47%, p < 0.05), which was obviously consistent with plasma melatonin (p < 0.001; r^2^ = 0.986), while there was no significance between 10 μg and control group (p = 0.559). These results indicated that the optimal injected concentration of PACAP6-38 was 20 μg per egg.

### Effect of PACAP6-38 on light-induced melatonin, GH and IGF-1 secretions

**Fig 2:**
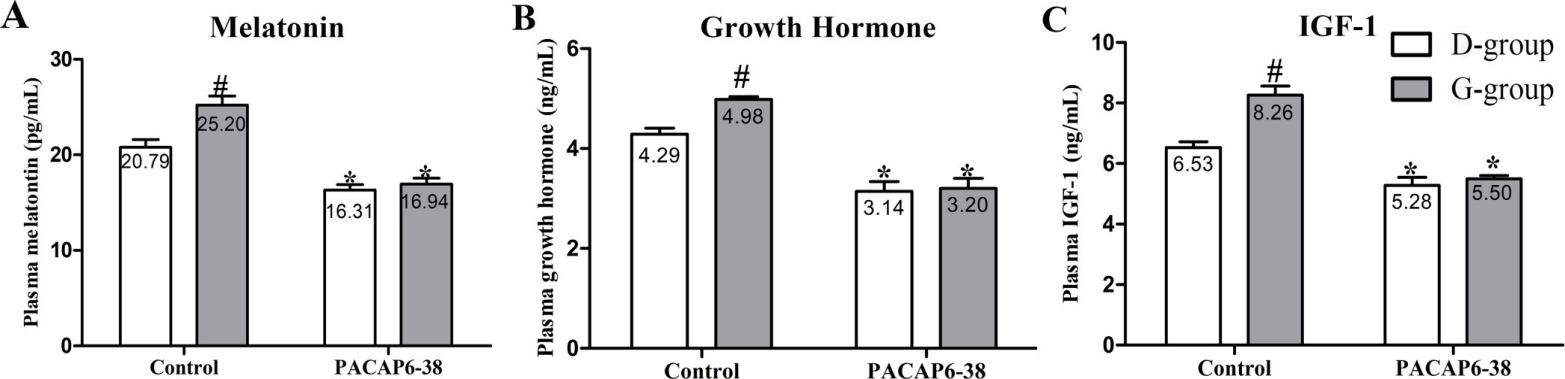
Effect of PACAP6-38 on plasma hormone contents in D-group and G-group. **(A)** melatonin, **(B)** growth hormone, and **(C)** IGF-1. Data are mean ± SEM. # p < 0.05 Vs. D-group control; * p < 0.05 Vs. different reagent treatment in the same light groups. Green light promotes the plasma contents of melatonin, growth hormone and IGF-1. After PACAP6-38 treatment, the difference in plasma contents of melatonin, growth hormone and IGF-1 in D-group and G-group disappeared. Meanwhile, the plasma hormone contents were significantly lower than control treatments.

As shown in Fig 2, the concentrations of plasma melatonin, GH and IGF-1 were affected by light illumination (melatonin: F = 11.020, p = 0.003; GH: F = 5.836, p = 0.025; IGF-1 = 17.84, p < 0.05) and PACAP6-38 injection (melatonin: F = 70.102, p < 0.05; GH: F = 88.087, p < 0.05; IGF-1 = 75.71, p < 0.05). The interaction of light illumination and PACAP6-38 injection has an effect on hormones (melatonin: F = 6.174, p = 0.022; GH: F = 4.010, p = 0.049; IGF-1 = 10.683, p = 0.004). In control saline treatment, the plasma melatonin level of G-group was higher by 21.24% than that of D-group (p = 0.003; Fig 2A). After PACAP6-38 treatment, the plasma melatonin level was decreased by 21.54% (p = 0.002) and 32.77% (p < 0.05) compared with the control treatment both in D-group and G-group, respectively. The PACAP6-38 treatment resulted in few light-induced differences in the plasma melatonin level between G-group and D-group (p = 0.934).

Similar to the concentration of melatonin, the plasma GH and IGF-1 of G-group were higher by 16.08% (p = 0.025) and 26.56% (p < 0.05) than those of D-group in the control saline treatment, respectively (Fig 2B and 2C). Compared with the control saline treatment, the PACAP6-38 decreased the plasma levels of GH and IGF-1 either in D-group (GH: 26.81%, p < 0.05; IGF-1: 19.15%, p = 0.005) or in G-group (GH: 35.68%, p < 0.05; IGF-1: 33.47%, p < 0.05), and no difference were observed between G-group and D-group in GH (p = 0.991) and IGF-1 (p = 0.908).

Pearson correlation analysis showed the positive correlations among the plasma melatonin, GH and IGF-1 concentrations (melatonin & GH, p = 0.001, r^2^ = 0.981; GH & IGF-1, p = 0.027, r^2^ = 0.947; melatonin & IGF-1, p = 0.004, r^2^ = 0.991), respectively.

### Effect of PACAP6-38 on light-induced skeletal muscle growth

Body weight, and pectoral and gastrocnemius muscle weight at P0 were affected by light illuminance (body weight: F = 4.522, p = 0.046; pectoral muscle weight: F = 0.001, p = 0.978; gastrocnemius muscle weight: F = 0.246, p = 0.625) and PACAP6-38 (body weight: F = 58.605, p < 0.05; pectoral muscle weight: F = 65.949, p < 0.05; gastrocnemius muscle weight: F = 38.133, p < 0.05; Table 2). The interaction of light illumination and PACAP6-38 injection has an effect on body weight (F = 1.377, p = 0.254), pectoral muscle weight (F = 5.541, p = 0.028) and gastrocnemius muscle weight (F = 2.213, p = 0.152). In control saline treatment, the body weight was higher by 10.36% in G-group than that of in D-group (p = 0.124). After PACAP6-38 treatment, the body weight was decreased by 20.34% (p < 0.05) and 25.11% (p < 0.05) compared with the control treatment both in D-group and G-group, respectively. However, there was no difference in body weight between G-group and D-group after PACAP6-38 treatment (p = 0.906).

**Table 2:** Effect of PACAP 6–38 on hatching weight, pectoral muscle weight and gastrocnemius muscle weight of chicken in D-group and G-group at P0.

Items	D-group	G-group
**Hatching weight**^**1**^ **(g)**		
Control (n = 20)	51.71±0.77	57.06±1.31
PACAP6-38 (n = 20)	41.21±1.97*	42.74±1.97*
**Pectoral muscle weight (0.1 g)**		
Control (n = 20)	12.72±0.23	13.44±0.14
PACAP6-38 (n = 20)	10.28±0.15*	10.28±0.52*
**Gastrocnemius muscle weight (0.1 g)**		
Control (n = 20)	6.36±0.13	6.50±0.1
PACAP6-38 (n = 20)	5.50±0.11*	5.14±0.32*

Data are mean ± SEM.

* p < 0.05 Vs. different reagent treatment in the same light groups.

^1^Average BW of all hatching chicks (both male and female) of each treatment group.

Similar to the change of body weight, the PACAP6-38 treatment decreased the index of pectoral and gastrocnemius muscles either in G-group (23.35% in pectoral muscle, p < 0.05; 21.63% in gastrocnemius muscle, p < 0.05) or in D-group (20.34% in pectoral muscle, p = 0.003; 13.39% in gastrocnemius muscle, p = 0.017) compared with the control saline treatment. No difference was observed between G-group and D-group (pectoral muscle, p = 0.358; gastrocnemius muscle, p = 0.512).

**Fig 3:**
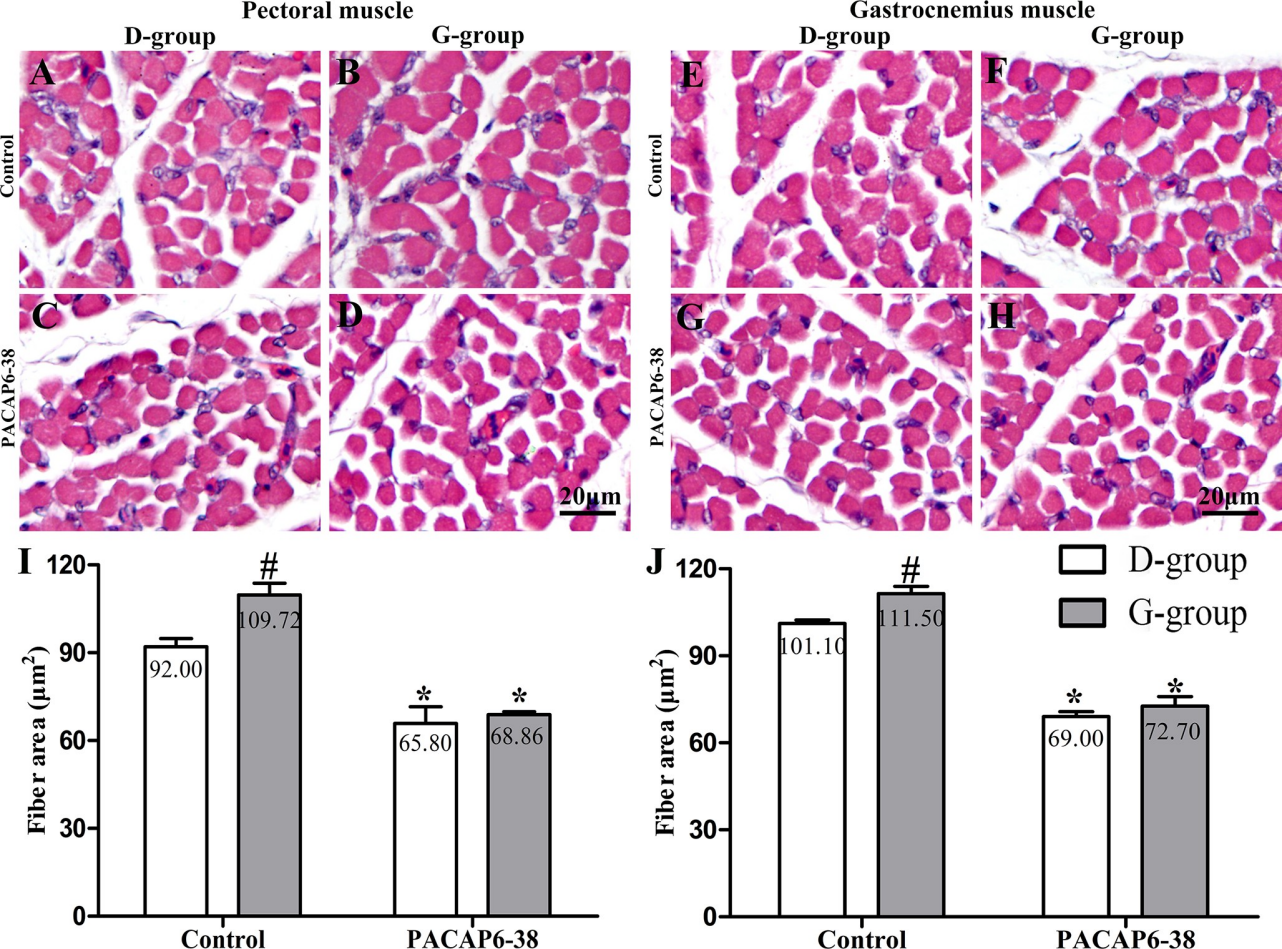
Effect of PACAP6-38 on myofiber areas of pectoral and gastrocnemius muscles in D-group and G-group. Representative images of pectoral (A-D) and gastrocnemius (E-H) muscles in different treatments of D-group and G-group. (I) and (J) are quantitation of fiber area. A, C, E, G: D-group; B, D, F, H: G-group; A, B, E, F: Control treatments; C, D, G, H: PACAP6-38 treatments. Data are mean ± SEM. # p < 0.05 Vs. D-group control; * p < 0.05 Vs. different reagent treatment in the same light groups. Green light significantly increased cross-sectional area of myofiber compared with D-group. PACAP6-38 treatment had significantly decreased myofiber cross-sectional area. And no significant difference was observed between D-group and G-group. Scale bar = 20 μm.

The myofiber cross-sectional area of pectoral and gastrocnemius muscle were affected by light (pectoral muscle: F = 5.950, p = 0.024; gastrocnemius muscle: F = 9.234, p = 0.006) and PACAP6-38 (pectoral muscle: F = 61.672, p < 0.05; gastrocnemius muscle: F = 233.718, p < 0.05). The interaction of light illumination and PACAP6-38 injection has an effect on the myofiber cross-sectional area of pectoral (F = 2.956, p < 0.05). In control saline treatment, the myofiber cross-sectional area of pectoral and gastrocnemius muscle in G-group were larger by 19.30% (p = 0.037) and 10.28% (p = 0.023) than those of in D-group (Fig 3). After PACAP6-38 treatment, the myofiber area was decreased by 29.32–31.75% (p = 0.000–0.002) and 34.80–45.30% (p < 0.05) compared to the control treatment both in D-group and G-group, respectively. The PACAP6-38 treatment resulted in few light-induced differences in pectoral (p = 0.956) and gastrocnemius muscle (p = 0.678).

### Effect of PACAP6-38 on light-induced PCNA-positive cell number

**Fig 4:**
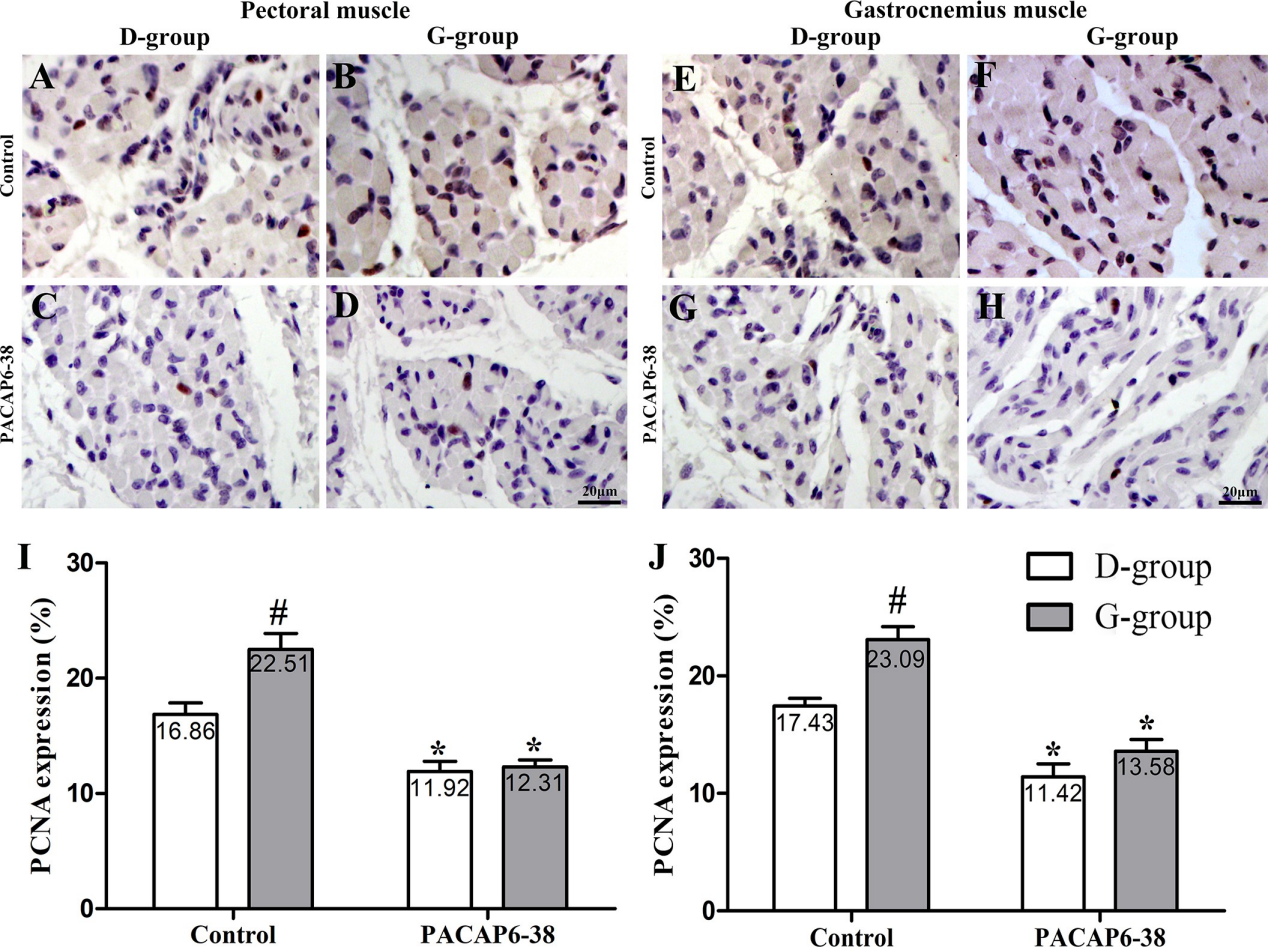
Effect of PACAP6-38 on the expression of PCNA in pectoral and gastrocnemius muscles in D-group and G-group. Characterization of PCNA-immunohistochemical staining in pectoral (A-D) and gastrocnemius (E-H) muscles in different treatments of D-group and G-group. (I) and (J) are quantitation of the expression of PCNA. A, C, E, G: D-group; B, D, F, H: G-group; A, B, E, F: Control treatments; C, D, G, H: PACAP6-38 treatments. Data are mean ± SEM. # p < 0.05 Vs. D-group control; * p < 0.05 Vs. different reagent treatment in the same light groups. Green light promotes the expression of PCNA in pectoral and gastrocnemius muscle compared with D-group. PACAP6-38 treatment had significantly decreased the expression of PCNA. And no significant difference was observed between D-group and G-group. Scale bar = 20 μm.

PCNA-immunohistochemical staining was used to evaluate the satellite cell proliferation of broilers exposed to green light during the incubation period. The positive cells displayed brownish yellow granules in the cell nucleus (Fig 4). The expression of PCNA in pectoral and gastrocnemius muscles were influenced by light illuminance (pectoral muscle: F = 9.019, p = 0.007; gastrocnemius muscle: F = 22.25, p < 0.05) and PACAP6-38 (pectoral muscle: F = 56.568, p < 0.05; gastrocnemius muscle: F = 88.07, p < 0.05). The interaction of light illumination and PACAP6-38 injection has an effect on the expression of PCNA in pectoral (F = 6.811, p = 0.017) and gastrocnemius muscles (F = 4.47, p = 0.047). In control saline treatment, the percentage of PCNA-positive cells was significantly higher in G-group than that of in D-group (33.51% in pectoral muscle, p = 0.004; 32.45% in gastrocnemius muscle, p < 0.05). However, the PACAP6-38 treatment resulted in few light-induced differences in the percentage of PCNA-positive cells between G-group and D-group (p > 0.05). After PACAP6-38 treatment, the percentage of PCNA-expressing cells was decreased by 29.32–34.49% (29.32% in pectoral muscle, p = 0.011; 34.49% in gastrocnemius muscle, p < 0.05) and 41.21–45.30% (p < 0.05) compared with the control treatment both in D-group and G-group, respectively.

### Effect of PACAP6-38 on light-induced satellite cell number

**Fig 5:**
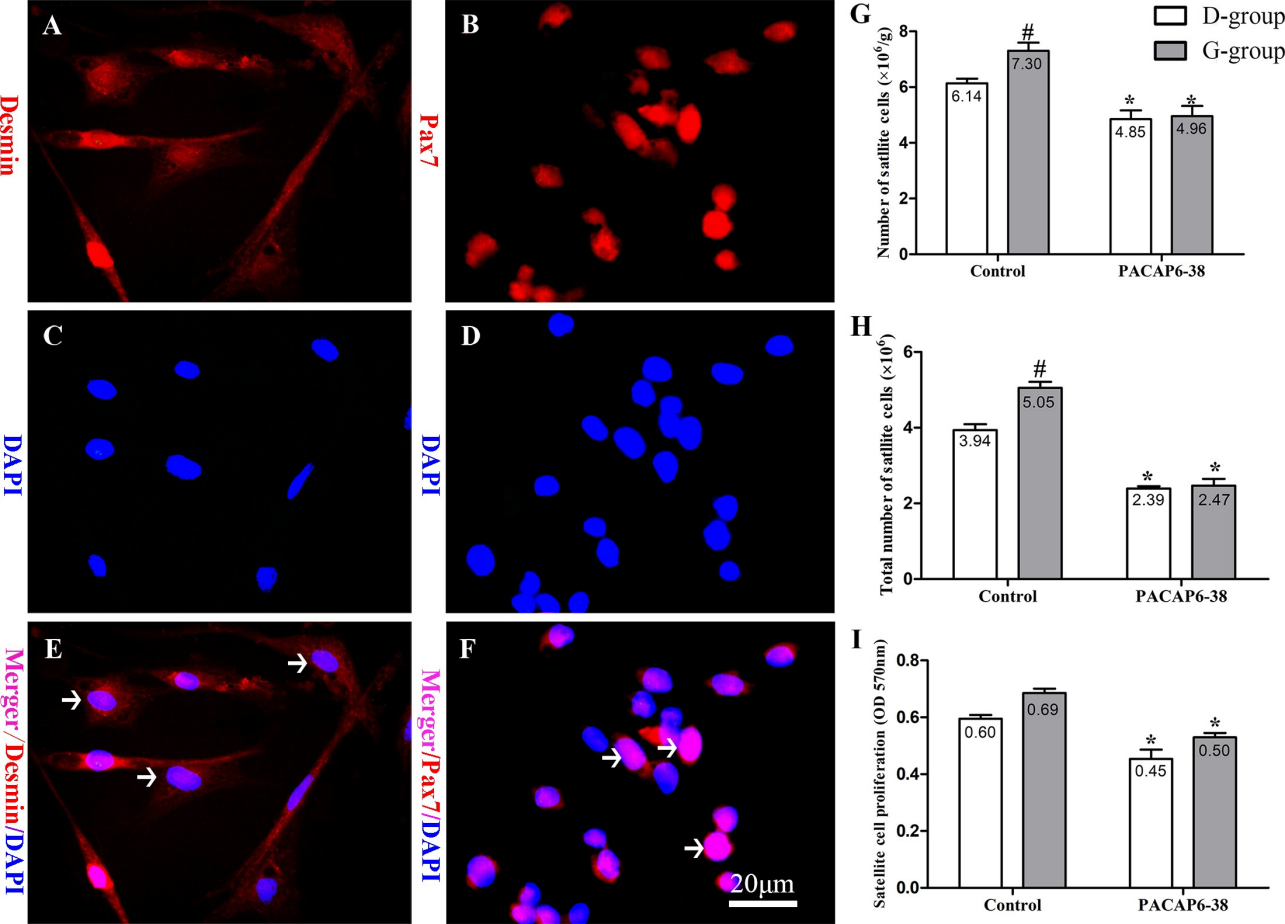
Effect of PACAP6-38 on number and proliferation of satellite cells in pectoral muscle. Identification of satellite cells by immunofluorescent staining of desmin (A: Desmin; C: DAPI; E: Merger) and Pax7 (B: Pax7; D: DAPI; F: Merger) in primary cultured cells. (G) Relative number of satellite cells. (H) Absolute number of satellite cells. (I) Proliferation of satellite cells. Data are mean ± SEM. # p < 0.05 Vs. D-group control; * p < 0.05 Vs. different reagent treatment in the same light groups. Desmin labeling the cellular structure (A, red), Pax7 expressed in cell nucleus (B, red), and nuclei are stained with DAPI (C and D, blue). The arrows indicate the positive cells (E, F). Green light increased the relative numbers, absolute numbers and proliferation of satellite cells compared with D-group. PACAP6-38 decreased the relative numbers, absolute numbers and proliferation of satellite cells, and the differences of these disappeared between D-group and G-group. Scale bar = 20 μm.

We isolated and cultured satellite cells in vitro from the skeletal muscle. Desmin labeled the cellular structure (Fig 5A, 5C and 5E), and the Pax7 expressed in cell nucleus (Fig 5B, 5D and 5F). The relative and absolute numbers of satellite cells from pectoral muscle were examined (Fig 5G and 5H).

As shown in Fig 5G, the skeletal muscle consisted of a high proportion of satellite cells to per gram muscle upon hatching in broilers. The relative number of the satellite cell was affected by light illumination (F = 4.749, p = 0.042) and PACAP6-38 (F = 38.461, p < 0.05). After PACAP6-38 treatment, the relative satellite cell proportion significantly decreased by 20.97–32.13% than that of control saline treatment (p < 0.05). In control saline treatment, the relative number of satellite cells of G-group was higher by 19.04% than that of in D-group (p = 0.048). The PACAP6-38 treatment resulted in few light-induced differences in the relative number of satellite cells between G-group and D-group (p = 0.994).

Effect of PACAP6-38 on light-induced absolute number of satellite cells was shown in Fig 5H. The absolute number of satellite cells was affected by light illumination (F = 13.124, p = 0.002) and PACAP6-38 (F = 157.133, p < 0.05). The interaction of light illumination and PACAP6-38 injection has an effect on the absolute number of satellite cells (F = 9.811, p = 0.005). In control saline treatment, the absolute number of satellite cells was higher by 28.26% in G-group than that of in D-group (p < 0.05). After PACAP6-38 treatment, the absolute number of satellite cells significantly decreased by 39.38% and 51.12% (p < 0.05) compared with the control treatment both in D-group and G-group. However, the difference of light-induced disappeared in PACAP6-38 treatment (p > 0.05). Furthermore, Pearson correlation analysis showed a positive correlation between the absolute and relative numbers of satellite cells (p = 0.002; r^2^ = 0.996).

Additionally, an MTT assay for cell proliferation was used to evaluate satellite cells mitotic activity (SCMA) (Fig 5I), which was influenced by light illumination (F = 5.605, p = 0.028) and PACAP6-38 (F = 33.830, p < 0.05). Green light stimulation increased the expression of the SCMA (15.41%, p = 0.138) compared with D-group in control saline treatment. And the SCMA in PACAP6-38 treatment was lower by 23.81 (p = 0.011) and 27.67% (p < 0.05) than that of in control treatment both in D-group and G-group, respectively.

### Effect of PACAP6-38 on light-induced myogenic gene expression

**Fig 6:**
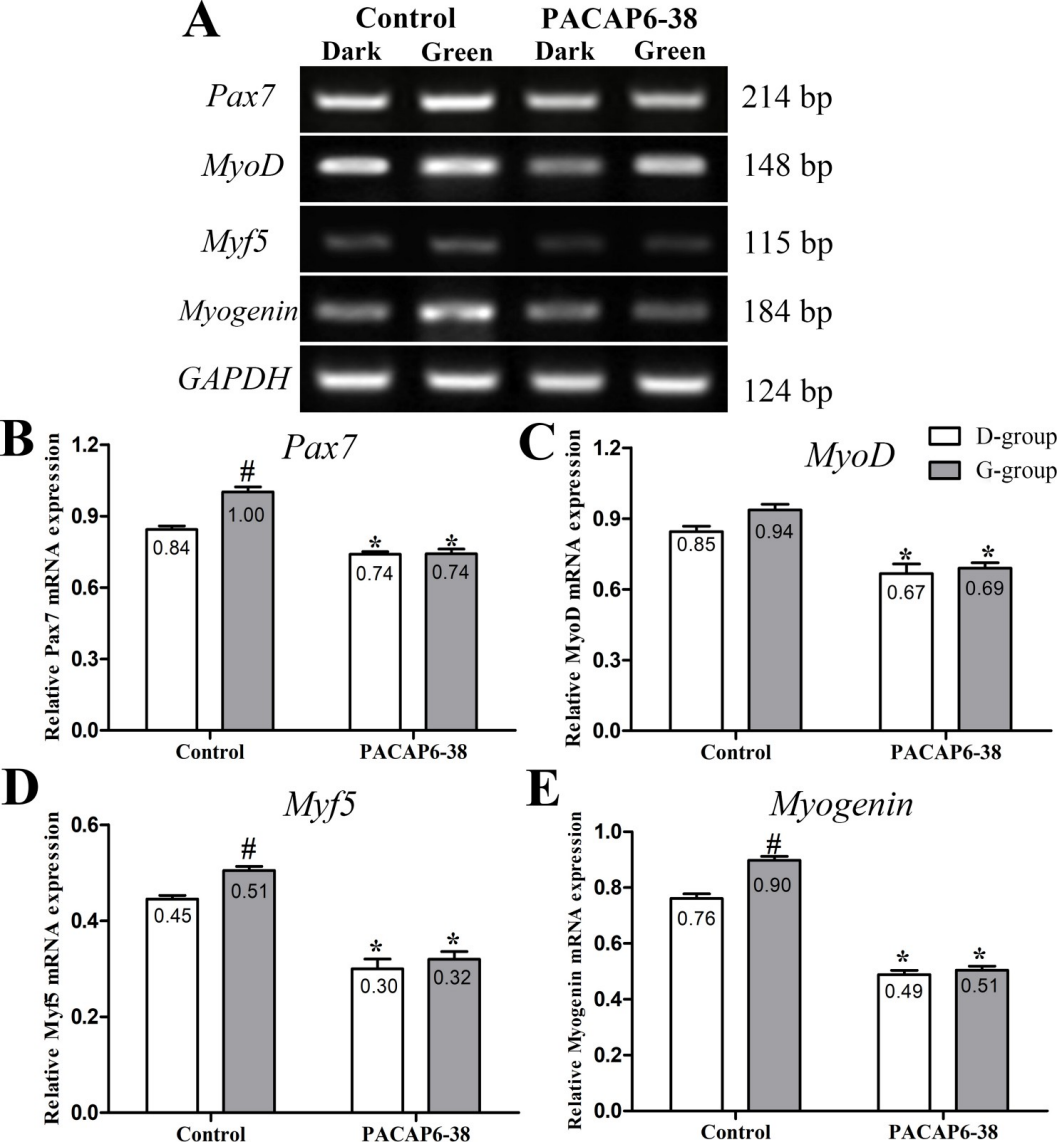
Effect of PACAP6-38 on myogenic genes expression in D-group and G-group. Electropherogram of RT-PCR for *Pax7*, *MyoD*, *Myf5* and *Myogenin* of skeletal muscle (A). Histograms demonstrating changes in *Pax7* (B), *MyoD* (C), *Myf5* (D) and *Myogenin* (E) expression by PACAP6-38 treatment both in D-group and G-group. Data are mean ± SEM. # p < 0.05 Vs. D-group control; * p < 0.05 Vs. different reagent treatment in the same light groups. Green light promoted the mRNA expression of *Pax7*, *MyoD*, *Myf5* and *Myogenin* compared with D-group. PACAP6-38 decreased the expression of myogenic genes, and no difference was observed between D-group and G-group.

The expression of *Pax7*, *MyoD*, *Myf5* and *Myogenin* mRNA level were affected by light illumination (*Pax7*: F = 21.27, p < 0.05; *MyoD*: F = 2.526, p = 0.128; *Myf5*: F = 7.708, p = 0.012; *Myogenin*: F = 31.99, p < 0.05) and PACAP6-38 (*Pax7*: F = 109.7, p < 0.05; *MyoD*: F = 47.690, p < 0.05; *Myf5*: F = 135.412, p < 0.05; *Myogenin*: F = 625.61, p < 0.05; Fig 6). The interaction of light illumination and PACAP6-38 injection has an effect on the expression of *Pax7* (F = 20.40, p < 0.05) and *Myogenin* (F = 18.26, p < 0.05). In control saline treatment, *Pax7*, *MyoD*, *Myf5* and *Myogenin* mRNA expression were higher (*Pax7*, 18.77%, p < 0.05; *MyoD*, 10.85%, p = 0.347; *Myf5*, 13.48%, p = 0.009; *Myogenin*, 17.94%, p < 0.05) in G-group than those of in D-group. After PACAP6-38 treatment, *Pax7*, *MyoD*, *Myf5* and *Myogenin* mRNA expression decreased (*Pax7*, 12.25–25.96%, p < 0.05; *MyoD*, 21.10–26.33%, p < 0.05; *Myf5*, 32.58–36.63%, p < 0.05; *Myogenin*, 35.89–43.78%, p < 0.05) compared with control saline treatment. The PACAP6-38 treatment resulted in few light-induced differences in *Pax7*, *MyoD*, *Myf5* and *Myogenin* mRNA expression (p > 0.05).

### Effect of PACAP6-38 on light-induced IGF-1 receptor mRNA expression

**Fig 7:**
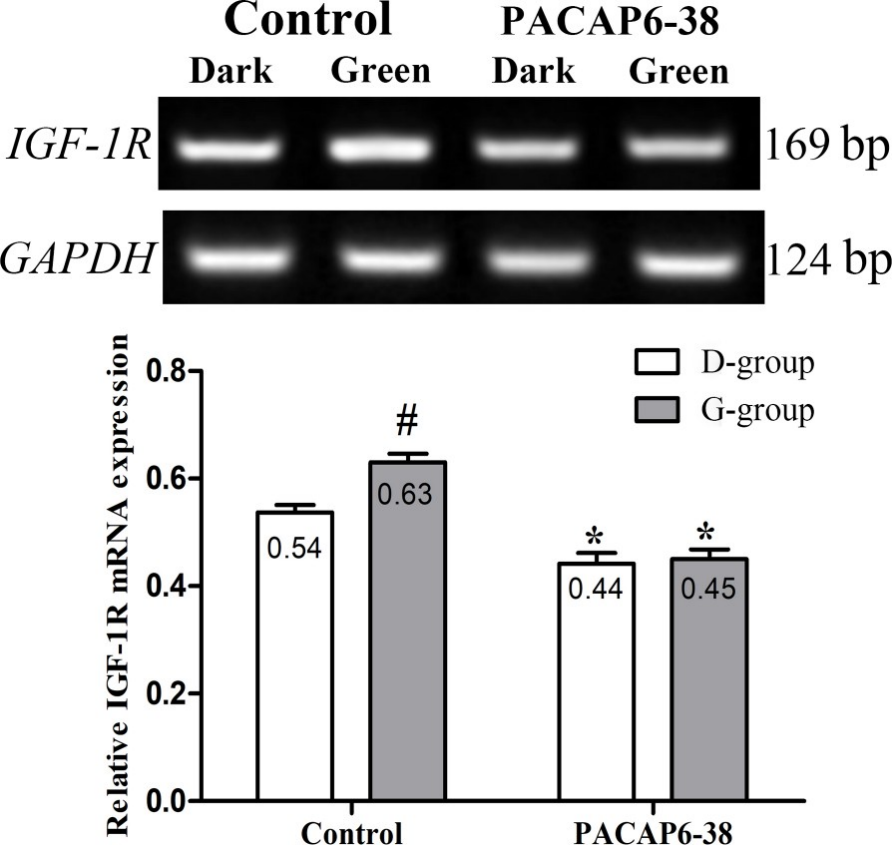
Effect of PACAP6-38 on *IGF-1R* expression of skeletal muscle in D-group and G-group. Data are mean ± SEM. # p < 0.05 Vs. D-group control; * p < 0.05 Vs. different reagent treatment in the same light groups. Green light promoted the mRNA expression of *IGF-1R* compared with D-group. PACAP 6–38 decreased the expression of *IGF-1R*, and no difference was observed between D-group and G-group.

The expression of *IGF-1 receptor* (*IGF-1R*) mRNA were detected in the pectoral muscle (Fig 7), which was influenced by light illumination (F = 8.468, p = 0.009) and PACAP6-38 (F = 61.959, p < 0.05). The interaction of light illumination and PACAP6-38 injection has an effect on the expression of *IGF-1R* (F = 5.919, p = 0.025). *IGF-1R* mRNA level of pectoral muscle in G-group was higher by 17.39% than that of in D-group in control saline treatment (p = 0.006). Compared with control saline treatment, the PACAP6-38 treatment decreased the expression of *IGF-1R* mRNA in D-group (17.70%, p = 0.005) and G-group (28.57%, p < 0.05), and resulted in few differences between G-group and D-group (p > 0.05).

## Discussion

Previous study have suggested that the light schedule [38], intensity [39] and wavelength [4, 6, 40] affect growth and development in birds. However, the number of muscle fibers remains constant after hatching, and the growth of skeletal muscle depends on the embryonic muscle development to a certain extent [41]. Therefore, it is important to study the effect of monochromatic light stimulation on growth and development during embryogenesis in broilers. Our results showed that the 0-day old broilers stimulated with monochromatic green light during embryogenesis had a heavier body weight (10.36%), a larger muscle weight (5.64% in pectoral muscle and 2.28% in gastrocnemius muscle), and a bigger myofiber cross-sectional area (pectoral muscle: 19.30%; gastrocnemius muscle: 10.28%) than those of D-group. Furthermore, our study demonstrated that monochromatic green light promoted satellite cell proliferation and increased the number of satellite cells in control saline treatment. These findings are consistent with our previous results, that is stimulation with monochromatic green light during incubation enhanced the skeletal muscle growth and satellite cell proliferation in late-embryogenesis (E15-E20) [10] and early stage (P1-P21) [8] of chicks. In this study, we further investigated the effects of monochromatic green light stimulation on the expression of *Pax7* and *Myogenic regulatory factors* (*MRFs*). We found that green light stimulation increased the mRNA expressions of *Pax7* (18.77%) and myogenic regulatory factors (*MyoD*, 10.85%, *Myf5*, 13.48% and *Myogenin*, 17.79%), which performed the satellite cell myogenic program. However, how does color of light affect the intracellular events, such as the expression of *Pax7* and *MRFs* in ovo should be further explored.

A more likely explanation is that monochromatic green light in embryogenesis indirectly affects myoblast proliferation via the endocrine system. IGF-1 and GH are important growth factors in the regulation of muscle development, which has been demonstrated both in avian [42] and mammals [43]. IGF-1 promotes embryonic myoblast proliferation in chickens by binding IGF-1R [44]. Exogenous rhIGF-1 could also affect the development of muscle in duck embryos [45]. The proliferation activity of the satellite cells harvested from the posthatched chick was increased by addition of a dose-depended rhIGF-1[6, 8]. Interestingly, in our study, the plasma IGF-1 and the expression of *IGF-1R* on skeletal muscle of G-group were higher by 26.49% and 16.67% than those of D-group (Figs 2C and 7), which was similar to the results of Liu and Bai et al. [6, 8]. Some reports suggested that light penetrate the skull and stimulate the pineal in bird [46, 47]. Light conditions affect the secretion of serotonin and melatonin in Japanese quail [38]. In our study, the concentration of melatonin and GH in plasma was higher by 21.21% and 16.08% in G-group than those of in D-group in saline control treatment (Fig 2A and 2B). These finding was similar to the result of Zhang et al., which green light stimulation increased the secretion of GH in young broilers [48]. Pearson correlation analysis showed that the positive correlations between the changes of the plasma melatonin, GH and IGF-1 concentrations. Meanwhile, all parameters positively associated with the positive rate of PCNA and the number of satellite cell. Moreover, green light significantly increased the number of PCNA and Pax7 positive cells compared with dark group at P0, whose expression trends were similar (data not shown). Our results suggested that the green light stimulation promoted the proliferation of satellite cell by melatonin, GH and IGF-1. Given these findings, we speculate that melatonin could mediate light-induced GH and IGF-1 secretion, and finally promoted satellite cell proliferation of chick.

In order to confirm the role of melatonin, we injected PACAP6-38 in ovo to inhibit the production of melatonin at E8. The plasma melatonin levels dropped significantly at P0 after PACAP6-38 treatment (lower by 32.77% in G-group and by 21.55% in D-group, Fig 2A). The PACAP6-38 treatment resulted in few light-induced differences in the plasma melatonin level between G-group and D-group. These results demonstrated that PACAP6-38 eliminated the light-induced differences in melatonin secretion. Meanwhile, the plasma GH and IGF-1 levels decreased by 26.80–35.74% and 19.30–33.41% after PACAP6-38 treatments, which indicated that melatonin mediated green light promoted GH secretion. It was supported by a previous report showed that melatonin mediates monochromatic light-induced GHRH expression in the hypothalamus and GH secretion in broilers [49]. Moreover, compared with the control treatment, the mRNA expression of myogenic factors had dropped (*Pax7*, 11.94–26.00%; *MyoD*, 21.18–25.81%; *Myf5*, 33.33–36.00% and *Myogenin*, 35.52–43.33%) with muscle development after PACAP6-38 treatment (Fig 6). Therefore, PACAP6-38 treatment declined the proliferation of satellite cells by 21.67–26.47% (Fig 5) and the development of myofiber size by 28.48–37.24% (f 3), respectively. Our results show that PACAP6-38 treatment caused changes in plasma melatonin, and long-lasting reduction in muscle growth. Moreover, the differences in all indicators related to muscle growth and hormone levels induced by color light were disappeared after PACAP6-38 treatment. These changes were significantly related to the plasma melatonin (p < 0.05). Therefore, PACAP6-38 treatment could eliminate the promotion of monochromatic green on satellite cell proliferation by inhibiting melatonin secretion.

Additionally, our previous studies confirmed that monochromatic green light enhanced the development of the chick embryo liver via an antioxidation pathway involving melatonin and melatonin receptor, Mel1c [35], and promoted IGF-1 secretion by the liver via JAK2/STAT3 in chick embryos [34]. Meanwhile, IGF-1 promote the satellite cell proliferation, differentiation and skeletal myotube hypertrophy by triggering the PI(3)K/Akt/mTOR and PI(3)K/Akt/GSK3 pathways and binding to IGF-1R [50]. Therefore, monochromatic green light accelerates the satellite cell proliferation by regulating GH-IGF-1 axis related to melatonin in chick embryo.

## Conclusion

Stimulation with monochromatic green light during incubation enhanced the secretion of melatonin and up-regulation of GH-IGF-1 axis. Subsequently, IGF-1 binds IGF-1R contributing to the proliferation of satellite cells and myofiber formation, involving the expression of *Pax7* and *myogenic regulatory factors*. However, these phenomena could be inhibited by PACAP6-38 treatment.

## Supporting information

S1 Data 20190425-Supporting information. (DOCX)

## References

[1] Halevy O, Biran I, Rozenboim I. Various light source treatments affect body and skeletal muscle growth by affecting skeletal muscle satellite cell proliferation in broilers. Comp Biochem Physiol A Mol Integr Physiol. 1998;120(2):317–23. 9773510 10.1016/s1095-6433(98)10032-6

[2] Lea RW, Richard-Yris MA, Sharp PJ. The effect of ovariectomy on concentrations of plasma prolactin and LH and parental behavior in the domestic fowl. Gen Comp Endocrinol. 1996;101(1):115–21. 10.1006/gcen.1996.0013 8713650

[3] Williams JB, Sharp PJ. A comparison of plasma progesterone and luteinizing hormone in growing hens from eight weeks of age to sexual maturity. J Endocrinol. 1977;75(3):447–8. 591858 10.1677/joe.0.0750447

[4] Cao J, Wang Z, Dong Y, Zhang Z, Li J, Li F, et al Effect of combinations of monochromatic lights on growth and productive performance of broilers. Poult Sci. 2012;91(12):3013–8. 10.3382/ps.2012-02413 23155007

[5] Sindhurakar A, Bradley NS. Light accelerates morphogenesis and acquisition of interlimb stepping in chick embryos. PLoS One. 2012;7(12):e51348 10.1371/journal.pone.0051348 23236480 PMC3516530

[6] Liu W, Wang Z, Chen Y. Effects of monochromatic light on developmental changes in satellite cell population of pectoral muscle in broilers during early posthatch period. Anat Rec. 2010;293(8):1315–24. 10.1002/ar.21174 20665810

[7] Ke Y, Liu W, Wang Z, Chen Y. Effects of monochromatic light on quality properties and antioxidation of meat in broilers. Poult Sci. 2011;90(11):2632–7. 10.3382/ps.2011-01523 22010251

[8] Bai X, Wang Y, Wang Z, Cao J, Dong Y, Chen Y. In ovo exposure to monochromatic lights affect posthatch muscle growth and satellite cell proliferation of chicks: role of IGF-1. Growth Factors. 2016;34(3–4):107–18. 10.1080/08977194.2016.1199553 27362374

[9] Rozenboim I, Piestun Y, Mobarkey N, Barak M, Hoyzman A, Halevy O. Monochromatic light stimuli during embryogenesis enhance embryo development and posthatch growth. Poult Sci. 2004;83(8):1413–9. 10.1093/ps/83.8.1413 15339018

[10] Wang Y, Bai X, Wang Z, Cao J, Dong Y, Dong Y, et al Various LED Wavelengths affected myofiber development and satellite cell proliferation of chick embryos via the IGF-1 signaling pathway. Photochem Photobiol. 2017;93(6):1492–501. 10.1111/php.12806 28708285

[11] Relaix F. Skeletal muscle progenitor cells: from embryo to adult. Cell Mol Life Sci. 2006;63(11):1221–5. 10.1007/s00018-006-6015-9 16699810 PMC11136415

[12] Wang YX, Rudnicki MA. Satellite cells, the engines of muscle repair. Nat Rev Mol Cell Biol. 2012;13(2):127–33. 10.1038/nrm3265 22186952

[13] Biressi S, Rando TA, editors. Heterogeneity in the muscle satellite cell population. Semin Cell Dev Biol; 2010;21(8):845–54. 10.1016/j.semcdb.2010.09.003 20849971 PMC2967620

[14] Halevy O, Hodik V, Mett A. The effects of growth hormone on avian skeletal muscle satellite cell proliferation and differentiation. Gen Comp Endocrinol. 1996;101(1):43–52. 10.1006/gcen.1996.0006 8713643

[15] Halevy O, Geyra A, Barak M, Uni Z, Sklan D. Early posthatch starvation decreases satellite cell proliferation and skeletal muscle growth in chicks. J Nutr. 2000;130(4):858–64. 10.1093/jn/130.4.858 10736342

[16] Punch VG, Jones AE, Rudnicki MA. Transcriptional networks that regulate muscle stem cell function. Wiley Interdiscip Rev Syst Biol Med. 2009;1(1):128–40. 10.1002/wsbm.11 20835986

[17] Dhawan J, Rando TA. Stem cells in postnatal myogenesis: molecular mechanisms of satellite cell quiescence, activation and replenishment. Trends Cell Biol. 2005;15(12):666–73. 10.1016/j.tcb.2005.10.007 16243526

[18] Bentzinger CF, von Maltzahn J, Rudnicki MA. Extrinsic regulation of satellite cell specification. Stem Cell Res Ther. 2010;1(3):27 10.1186/scrt27 20804582 PMC2941119

[19] Motohashi N, Asakura A. Muscle satellite cell heterogeneity and self-renewal. Front Cell Dev Biol. 2014;2:1 10.3389/fcell.2014.00001 25364710 PMC4206996

[20] Miyata A, Arimura A, Dahl RR, Minamino N, Uehara A, Jiang L, et al Isolation of a novel 38 residue-hypothalamic polypeptide which stimulates adenylate cyclase in pituitary cells. Biochem Biophys Res Commun. 1989;164(1):567–74. 2803320 10.1016/0006-291x(89)91757-9

[21] Vaudry D, Falluel-Morel A, Bourgault S, Basille M, Burel D, Wurtz O, et al Pituitary adenylate cyclase-activating polypeptide and its receptors: 20 years after the discovery. Pharmacol Rev. 2009;61(3):283–357. 10.1124/pr.109.001370 19805477

[22] Peeters K, Langouche L, Vandesande F, Darras V, Berghman L. Effects of Pituitary Adenylate Cyclase‐Activating Polypeptide (PACAP) on cAMP Formation and Growth Hormone Release from Chicken Anterior Pituitary Cellsa. Ann N Y Acad Sci. 1998;865(1):471–4. 9928052 10.1111/j.1749-6632.1998.tb11218.x

[23] Fukuchi M, Kuwana Y, Tabuchi A, Tsuda M. Balance between cAMP and Ca^2+^ signals regulates expression levels of pituitary adenylate cyclase-activating polypeptide gene in neurons. Genes to cells. 2016;21:921–9. 10.1111/gtc.12393 27383213

[24] Fukuhara C, Inouye SI, Matsumoto Y, Tsujimoto G, Aoki K, Masuo Y. Pituitary adenylate cyclase-activating polypeptide rhythm in the rat pineal gland. Neurosci Lett. 1998;241(2–3):115–8. 9507934 10.1016/s0304-3940(98)00041-x

[25] Darvish N, Russell JT. Neurotransmitter-induced novel modulation of a nonselective cation channel by a cAMP-dependent mechanism in rat pineal cells. J Neurophysiol. 1998;79(5):2546–56. 10.1152/jn.1998.79.5.2546 9582227

[26] Csernus V, Józsa R, Reglö D, Hollósy T, Somogyvári-Vigh A, Arimura A. The effect of PACAP on rhythmic melatonin release of avian pineals. Gen Comp Endocrinol. 2004;135(1):62–9. 14644645 10.1016/s0016-6480(03)00284-3

[27] Faluhelyi N, Reglodi D, Csernus V. The effects of PACAP and VIP on the in vitro melatonin secretion from the embryonic chicken pineal gland. Ann N Y Acad Sci. 2006;1070:271–5. 10.1196/annals.1317.025 16888177

[28] Nakahara K, Abe Y, Murakami T, Shiota K, Murakami N. Pituitary adenylate cyclase-activating polypeptide (PACAP) is involved in melatonin release via the specific receptor PACAP-r1, but not in the circadian oscillator, in chick pineal cells. Brain Res. 2002;939(1–2):19–25. 12020847 10.1016/s0006-8993(02)02538-6

[29] Józsa R, Somogyvári-Vigh A, Reglödi D, Hollosy T, Arimura A. Distribution and daily variations of PACAP in the chicken brain. Peptides. 2001;22(9):1371–7. 11514017 10.1016/s0196-9781(01)00477-6

[30] Erhardt NM, Fradinger EA, Cervini LA, Rivier JE, Sherwood NM. Early expression of pituitary adenylate cyclase-activating polypeptide and activation of its receptor in chick neuroblasts. Endocrinology. 2001;142(4):1616–25. 10.1210/endo.142.4.8105 11250943

[31] Fukuhara C, Suzuki N, Matsumoto Y, Nakayama Y, Aoki K, Tsujimoto G, et al Day-night variation of pituitary adenylate cyclase-activating polypeptide (PACAP) level in the rat suprachiasmatic nucleus. Neurosci Lett. 1997;229(1):49–52. 9224799 10.1016/s0304-3940(97)00415-1

[32] Hollósy T, Józsa R, Jakab B, Németh J, Lengvári I, Reglődi D. Effects of in ovo treatment with PACAP antagonist on general activity, motor and social behavior of chickens. Regul Pept. 2004;123(1):99–106. 15518899 10.1016/j.regpep.2004.05.018

[33] Shen S, Gehlert DR, Collier DA. PACAP and PAC1 receptor in brain development and behavior. Neuropeptides. 2013;47(6):421–30. 10.1016/j.npep.2013.10.005 24220567

[34] Wang T, Dong Y, Wang Z, Cao J, Chen Y. Secretion pathway of liver IGF-1 via JAK2/STAT3 in chick embryo under the monochromatic light. Growth Factors. 2016;34(1–2):51–63. 10.3109/08977194.2016.1170679 27144424

[35] Wang T, Wang Z, Cao J, Dong Y, Chen Y. Monochromatic light affects the development of chick embryo liver via an anti-oxidation pathway involving melatonin and the melatonin receptor Mel1c. Can J Anim Sci. 2014;93(3):391–400.

[36] Arimura A, Somogyvari A, Weill C, Fiore R, Tatsuno I, Bay V, et al PACAP functions as a neurotrophic factora. Ann N Y Acad Sci. 1994;739(1):228–43. 7726997 10.1111/j.1749-6632.1994.tb19825.x

[37] Grohmann M, Foulstone E, Welsh G, Holly J, Shield J, Crowne E, et al Isolation and validation of human prepubertal skeletal muscle cells: maturation and metabolic effects of IGF-I, IGFBP-3 and TNFα. J Physiol. 2005;568(1):229–42. 16081485 10.1113/jphysiol.2005.093906 PMC1474756

[38] Moore CB, Siopes TD. Effects of lighting conditions and melatonin supplementation on the cellular and humoral immune responses in Japanese quail Coturnix coturnix japonica. Gen Comp Endocrinol. 2000;119(1):95–104. 10.1006/gcen.2000.7496 10882554

[39] Yahav S, Hurwitz S, Rozenboim I. The effect of light intensity on growth and development of turkey toms. Br Poult Sci. 2000;41(1):101–6. 10.1080/00071660086484 10821531

[40] Kim M, Parvin R, Mushtaq M, Hwangbo J, Kim J, Na J, et al Growth performance and hematological traits of broiler chickens reared under assorted monochromatic light sources. Poult Sci. 2013;92(6):1461–6. 10.3382/ps.2012-02945 23687140

[41] Smith JH. Relation of body size to muscle cell size and number in the chicken. Poult Sci. 1963; 42(2):283–90.

[42] Duclos MJ. Insulin-like growth factor-I (IGF-1) mRNA levels and chicken muscle growth. J Physiol Pharmacol. 2005;56 Suppl 3:25–35. 16077194

[43] Shavlakadze T, Chai J, Maley K, Cozens G, Grounds G, Winn N, et al A growth stimulus is needed for IGF-1 to induce skeletal muscle hypertrophy in vivo. J Cell Sci. 2010;123(Pt6):960–71. 20179101 10.1242/jcs.061119

[44] Yu M, Wang H, Xu Y, Yu D, Li D, Liu X, et al Insulin-like growth factor-1 (IGF-1) promotes myoblast proliferation and skeletal muscle growth of embryonic chickens via the PI3K/Akt signalling pathway. Cell Biol Int. 2015;39(8):910–22. 10.1002/cbin.10466 25808997

[45] Liu H, Wang J, Chen X, Zhang R, Yu H, Jin H, et al In ovo administration of rhIGF-1 to duck eggs affects the expression of myogenic transcription factors and muscle mass during late embryo development. J Appl Physiol. 2011;111(6):1789–97. 10.1152/japplphysiol.00551.2011 21885804

[46] Binkley SA, Riebman JB, Reilly KB. The pineal gland: a biological clock in vitro. Science. 1978;202(4374):1198–200. 214852 10.1126/science.214852

[47] Deguchi T. A circadian oscillator in cultured cells of chicken pineal gland. Nature. 1979;282:94–6. 503196 10.1038/282094a0

[48] Musaro A, McCullagh KJ, Naya FJ, Olson EN, Rosenthal N. IGF-1 induces skeletal myocyte hypertrophy through calcineurin in association with GATA-2 and NF-ATc1. Nature. 1999;400(6744):581–5. 10.1038/23060 10448862

[49] Zhang L, Cao J, Wang Z, Dong Y, Chen Y. Melatonin modulates monochromatic light-induced GHRH expression in the hypothalamus and GH secretion in chicks. Acta Histochem. 2016;118(3):286–92. 10.1016/j.acthis.2016.02.005 26948666

[50] Rommel C, Bodine SC, Clarke BA, Rossman R, Nunez L, Stitt TN, et al Mediation of IGF-1-induced skeletal myotube hypertrophy by PI (3) K/Akt/mTOR and PI (3) K/Akt/GSK3 pathways. Nat Cell Biol. 2001;3(11):1009–13. 10.1038/ncb1101-1009 11715022

