# Supplementary material for: Melatonin mediates monochromatic green light-induced satellite cell proliferation and muscle growth in chick embryo
Source: PLoS One. 2019 May 6;14(5):e0216392. doi: 10.1371/journal.pone.0216392 (PMC6502336; doi:10.1371/journal.pone.0216392)
Supplement: S1 Data — (DOCX) [file pone.0216392.s001.docx]

**Supporting information**

**S1 Table. The data of determination of PACAP6-38 injected dose for Fig.1.**

|  | **0 μg** | **10 μg** | **20 μg** | **40 μg** | **60 μg** |
| --- | --- | --- | --- | --- | --- |
| **Plasma melatonin (pg/mL)** | 22.51 | 21.74 | 16.76 | 18.06 | 15.28 |
|  | 22.63 | 21.67 | 17.94 | 18.11 | 15.37 |
|  | 22.68 | 21.68 | 17.96 | 18.14 | 15.40 |
|  | 20.53 | 18.58 | 14.98 | 14.19 | 13.61 |
|  | 20.62 | 18.64 | 15.09 | 14.31 | 13.67 |
|  | 20.64 | 18.67 | 15.12 | 14.35 | 13.72 |
| **Fiber area (μm^2^)** | 92.97 | 88.20 | 60.61 | 44.68 | 39.34 |
|  | 91.98 | 77.08 | 76.67 | 52.95 | 61.51 |
|  | 86.65 | 73.98 | 90.23 | 43.93 | 61.32 |
|  | 85.63 | 81.86 | 54.25 | 74.58 | 54.84 |
|  | 96.40 | 93.17 | 49.64 | 92.56 | 59.77 |
|  | 98.38 | 71.55 | 63.43 | 67.96 | 68.09 |

**S2 Table. The data of effect of PACAP6-38 on plasma melatonin, growth hormone and IGF-1 secretions for Fig.2.**

|  | **D-control** | **G-control** | **D-PACAP6-38** | **G-PACAP6-38** |
| --- | --- | --- | --- | --- |
| **Melatonin (pg/mL)** | 22.53 | 24.30 | 16.76 | 17.08 |
|  | 22.63 | 24.47 | 17.94 | 17.10 |
|  | 22.68 | 24.49 | 17.96 | 17.10 |
|  | 18.84 | 25.62 | 14.98 | 15.37 |
|  | 18.99 | 29.63 | 15.09 | 15.50 |
|  | 19.05 | 22.70 | 15.12 | 19.51 |
| **Growth hormone (ng/mL)** | 4.02 | 4.76 | 3.71 | 3.33 |
|  | 4.21 | 5.12 | 2.84 | 2.91 |
|  | 4.64 | 5.06 | 3.37 | 3.37 |
|  | 4.20 | 5.10 | 2.34 | 3.87 |
|  | 4.66 | 4.98 | 3.38 | 3.33 |
|  | 4.01 | 4.86 | 3.20 | 2.41 |
| **IGF-1 (ng/mL)** | 6.25 | 9.45 | 5.39 | 5.62 |
|  | 7.14 | 8.39 | 5.77 | 5.83 |
|  | 6.20 | 8.37 | 5.80 | 5.61 |
|  | 7.14 | 8.11 | 4.45 | 5.57 |
|  | 6.22 | 7.14 | 5.79 | 5.19 |
|  | 6.21 | 8.10 | 4.46 | 5.15 |

**S3 Table. The date of effect of PACAP6-38 on myofiber areas of pectoral and gastrocnemius muscles for Fig.3.**

|  | **control-D** | **control-G** | **D-PACAP6-38** | **G-PACAP6-38** |
| --- | --- | --- | --- | --- |
| **Myofiber areas of pectoral muscle (μm^2^)** | 93.79 | 101.24 | 60.61 | 71.57 |
|  | 86.13 | 102.73 | 63.48 | 67.03 |
|  | 96.90 | 124.13 | 80.75 | 66.70 |
|  | 91.97 | 119.91 | 92.43 | 66.92 |
|  | 85.63 | 106.96 | 47.79 | 71.09 |
|  | 97.40 | 103.37 | 49.64 | 69.83 |
| **Myofiber areas of gastrocnemius muscle (μm^2^)** | 98.78 | 113.60 | 66.81 | 61.53 |
|  | 101.26 | 112.26 | 61.86 | 65.50 |
|  | 104.91 | 115.71 | 70.31 | 74.44 |
|  | 99.41 | 104.91 | 70.41 | 83.87 |
|  | 104.55 | 119.00 | 74.46 | 76.55 |
|  | 97.69 | 103.50 | 70.17 | 74.30 |

**S4 Table. The date of effect of PACAP6-38 on PCNA expression in pectoral and gastrocnemius muscles for Fig.4.**

|  | **control-D** | **control-G** | **D-PACAP6-38** | **G-PACAP6-38** |
| --- | --- | --- | --- | --- |
| **PCNA expression in pectoral muscle (%)** | 14.29 | 25.00 | 10.53 | 12.00 |
|  | 17.78 | 18.75 | 13.54 | 14.29 |
|  | 21.28 | 27.27 | 10.43 | 10.53 |
|  | 16.67 | 22.22 | 15.56 | 13.64 |
|  | 16.00 | 18.75 | 10.53 | 11.11 |
|  | 15.15 | 23.08 | 10.92 | 12.31 |
| **PCNA expression in gastrocnemius (%)** | 17.65 | 25.93 | 11.76 | 16.67 |
|  | 16.67 | 22.22 | 12.50 | 13.33 |
|  | 20.00 | 19.35 | 13.89 | 11.11 |
|  | 17.68 | 24.14 | 7.41 | 15.00 |
|  | 15.00 | 23.81 | 11.54 | 11.76 |
|  | 17.62 | 23.09 | 11.42 | 13.57 |

**S5 Table. The date of effect of PACAP6-38 on number and proliferation of satellite cells in pectoral muscle for Fig.5.**

|  | **D-control** | **G-control** | **D-PACAP6-38** | **G-PACAP6-38** |
| --- | --- | --- | --- | --- |
| **The relative number of the satellite cells (×10^6^/g)** | 6.38 | 6.59 | 3.73 | 4.44 |
|  | 5.97 | 8.06 | 4.93 | 5.84 |
|  | 6.57 | 7.46 | 4.50 | 4.43 |
|  | 5.80 | 8.21 | 5.87 | 4.35 |
|  | 6.50 | 6.72 | 4.55 | 4.34 |
|  | 5.59 | 6.78 | 5.51 | 6.34 |
| **The absolute number of satellite cells (×10^6^)** | 4.44 | 4.50 | 2.13 | 1.94 |
|  | 4.25 | 5.44 | 2.38 | 3.06 |
|  | 3.94 | 5.31 | 2.50 | 3.13 |
|  | 3.13 | 5.25 | 2.44 | 2.00 |
|  | 3.87 | 4.63 | 2.63 | 2.44 |
|  | 4.01 | 5.19 | 2.25 | 2.25 |
| **MTT assay** | 0.59 | 0.76 | 0.44 | 0.47 |
|  | 0.60 | 0.69 | 0.45 | 0.39 |
|  | 0.54 | 0.72 | 0.61 | 0.38 |
|  | 0.59 | 0.67 | 0.44 | 0.57 |
|  | 0.64 | 0.62 | 0.40 | 0.60 |
|  | 0.61 | 0.66 | 0.38 | 0.57 |

**S6 Table. The date of effect of PACAP6-38 on myogenic genes expression for Fig.6.**

|  | **D-control** | **G-control** | **D-PACAP6-38** | **G-PACAP6-38** |
| --- | --- | --- | --- | --- |
| **Relative Pax7 mRNA expression** | 0.91 | 1.01 | 0.75 | 0.72 |
|  | 0.86 | 0.91 | 0.70 | 0.71 |
|  | 0.82 | 1.00 | 0.77 | 0.76 |
|  | 0.80 | 1.07 | 0.72 | 0.83 |
|  | 0.84 | 1.02 | 0.76 | 0.69 |
|  | 0.83 | 1.00 | 0.74 | 0.74 |
| **Relative MyoD mRNA expression** | 0.85 | 1.00 | 0.75 | 0.71 |
|  | 0.83 | 0.89 | 0.63 | 0.65 |
|  | 0.76 | 0.91 | 0.51 | 0.67 |
|  | 0.90 | 0.97 | 0.69 | 0.72 |
|  | 0.82 | 0.86 | 0.63 | 0.62 |
|  | 0.91 | 0.99 | 0.79 | 0.77 |
| **Relative Myf5 mRNA expression** | 0.45 | 0.54 | 0.26 | 0.31 |
|  | 0.42 | 0.51 | 0.31 | 0.36 |
|  | 0.46 | 0.51 | 0.30 | 0.37 |
|  | 0.44 | 0.49 | 0.25 | 0.27 |
|  | 0.47 | 0.50 | 0.39 | 0.29 |
|  | 0.43 | 0.48 | 0.29 | 0.32 |
| **Relative Myogenin mRNA expression** | 0.83 | 0.88 | 0.56 | 0.49 |
|  | 0.78 | 0.90 | 0.48 | 0.56 |
|  | 0.74 | 0.92 | 0.47 | 0.52 |
|  | 0.71 | 0.95 | 0.48 | 0.48 |
|  | 0.76 | 0.85 | 0.45 | 0.47 |
|  | 0.75 | 0.89 | 0.49 | 0.51 |

**S7 Table. The date of effect of PACAP6-38 on IGF-1R expression of skeletal muscle for Fig.7.**

| **control-D** | **control-G** | **D-PACAP6-38** | **G-PACAP6-38** |
| --- | --- | --- | --- |
| **0.55** | **0.67** | **0.50** | **0.41** |
| **0.59** | **0.56** | **0.45** | **0.48** |
| **0.48** | **0.65** | **0.48** | **0.44** |
| **0.53** | **0.66** | **0.36** | **0.52** |
| **0.54** | **0.63** | **0.42** | **0.40** |
| **0.53** | **0.61** | **0.44** | **0.45** |

**S8 Table. The date of effect of PACAP6-38 on hatching weight, pectoral muscle weight and gastrocnemius muscle weigh for Table 2.**

|  | **D-control** | **G-control** | **D-PACAP6-38** | **G-PACAP6-38** |
| --- | --- | --- | --- | --- |
| **Hatching weight (g)** | 50.24 | 51.57 | 42.15 | 44.15 |
|  | 54.18 | 53.94 | 41.91 | 47.4 |
|  | 52.06 | 61.87 | 33.08 | 43.45 |
|  | 49.96 | 56.91 | 50.46 | 39.89 |
|  | 49.41 | 59.55 | 41.58 | 43.65 |
|  | 54.39 | 58.53 | 37.95 | 37.87 |
| **Pectoral muscle weight (g)** | 1.23 | 1.34 | 1.14 | 1.24 |
|  | 1.34 | 1.35 | 1.10 | 0.92 |
|  | 1.24 | 1.40 | 1.08 | 0.98 |
|  | 1.21 | 1.30 | 1.04 | 0.90 |
|  | 1.33 | 1.32 | 1.13 | 1.10 |
|  | 1.27 | 1.34 | 1.11 | 1.03 |
| **Gastrocnemius muscle weight (g)** | 0.59 | 0.66 | 0.54 | 0.43 |
|  | 0.64 | 0.61 | 0.59 | 0.59 |
|  | 0.61 | 0.66 | 0.51 | 0.58 |
|  | 0.66 | 0.65 | 0.55 | 0.44 |
|  | 0.65 | 0.67 | 0.56 | 0.58 |
|  | 0.67 | 0.68 | 0.55 | 0.46 |
